# High fat diet deviates PtC-specific B1 B cell phagocytosis in obese mice

**DOI:** 10.1002/iid3.41

**Published:** 2014-12-15

**Authors:** Hung Vo, Joanna Chiu, Danielle Allaimo, Changchuin Mao, Yaqi Wang, Yuefei Gong, Hooisweng Ow, Tyrone Porter, Xuemei Zhong

**Affiliations:** 1 Hematology Oncology Section, Department of Medicine, Boston University Medical CenterBoston, MA; 2 Department of Biomedical Engineering, Boston UniversityBoston, MA; 3 Hybrid Silica TechnologiesCambridge, MA; 4 Department of Mechanical Engineering, Boston UniversityBoston, MA

**Keywords:** B1 B cell, high fat diet, immune deviation, obesity, phagocytosis, phosphatidylcholine

## Abstract

Phagocytosis had been attributed predominantly to “professional” phagocytes such as macrophages, which play critical roles in adipose tissue inflammation. However, recently, macrophage-like phagocytic activity has been reported in B1 B lymphocytes. Intrigued by the long-established correlation between high fat diet (HFD)-induced obesity and immune dysfunction, we investigated how HFD affects B1 B cell phagocytosis. A significant number of B1 B cells recognize phosphatidylcholine (PtC), a common phospholipid component of cell membrane. We report here that unlike macrophages, B1 B cells have a unique PtC-specific phagocytic function. In the presence of both PtC-coated and non-PtC control fluorescent nano-particles, B1 B cells from healthy lean mice selectively engulfed PtC-coated beads, whereas B1 B cells from HFD-fed obese mice non-discriminately phagocytosed both PtC-coated and control beads. Morphologically, B1 B cells from obese mice resembled macrophages, displaying enlarged cytosol and engulfed more beads. Our study suggests for the first time that HFD can affect B1 B cell phagocytosis, substantiating the link of HFD-induced obesity and immune deviation.

## Introduction

Phagocytosis is an essential cellular function for both immune defense and tissue homeostasis 1–3. Phagocytosis was attributed predominantly to “professional” phagocytes such as macrophages 4–6. However, recent reports show that B1 B lymphocytes possess phagocytic capacity and that they can present phagocytosed antigens to CD4 T cells 7–12. Adoptive transfer of B1 B cells into macrophage-deficient op/op(−/−) mice restored the macrophage pool 13,14. This supports the notion proposed by Borrello and Phipps early in 1996 that phagocytic CD5^+^ B cells are not a result of malignancy, but rather evidence of B cell-to-macrophage trans-lineage differentiation 15.

B1 B cells recognize phosphorylcholine (PC) and phosphatidylcholine (PtC), where PC is the polar head group of PtC, a major component of cell membrane phospholipids 16,17. To test if HFD affects B1 B cell PtC-specific phagocytosis, we investigated their phagocytic activity with PtC-coated fluorescent particles. Here, we report that B1 B cells exclusively internalize PtC-coated beads but not control beads, whereas macrophages have no such preference. More importantly, we show that HFD could significantly influence B1 B cell phagocytosis by deviating from PtC-specific phagocytosis to non-specific phagocytosis. As PtC-reactivity of B1 B cell is essential for immune defense and tissue homeostasis, such HFD-induced immune deviation could result in immune deficiencies in obese patients.

## Result and Discussion

### Peritoneal B1 B cells are distinct from macrophages in their specificity of phagocytosis

Several groups recently reported that murine B1 B cells have phagocytic and microbicidal abilities 10,11,18,19. To further investigate these novel functions of B1 B cells, we generated PtC-coated fluorescent beads. Green-fluorescent beads have PtC molecules attached to their surfaces and red-fluorescent beads with no surface PtC were used as control. As mentioned above, PtC is a major component of the cell membrane and a classical B1 B cell self-antigen. Thus, these particles may mimic the membrane of PtC exposed apoptotic cells or the membrane of bioactive microvesicles 20.

**Figure 1:**
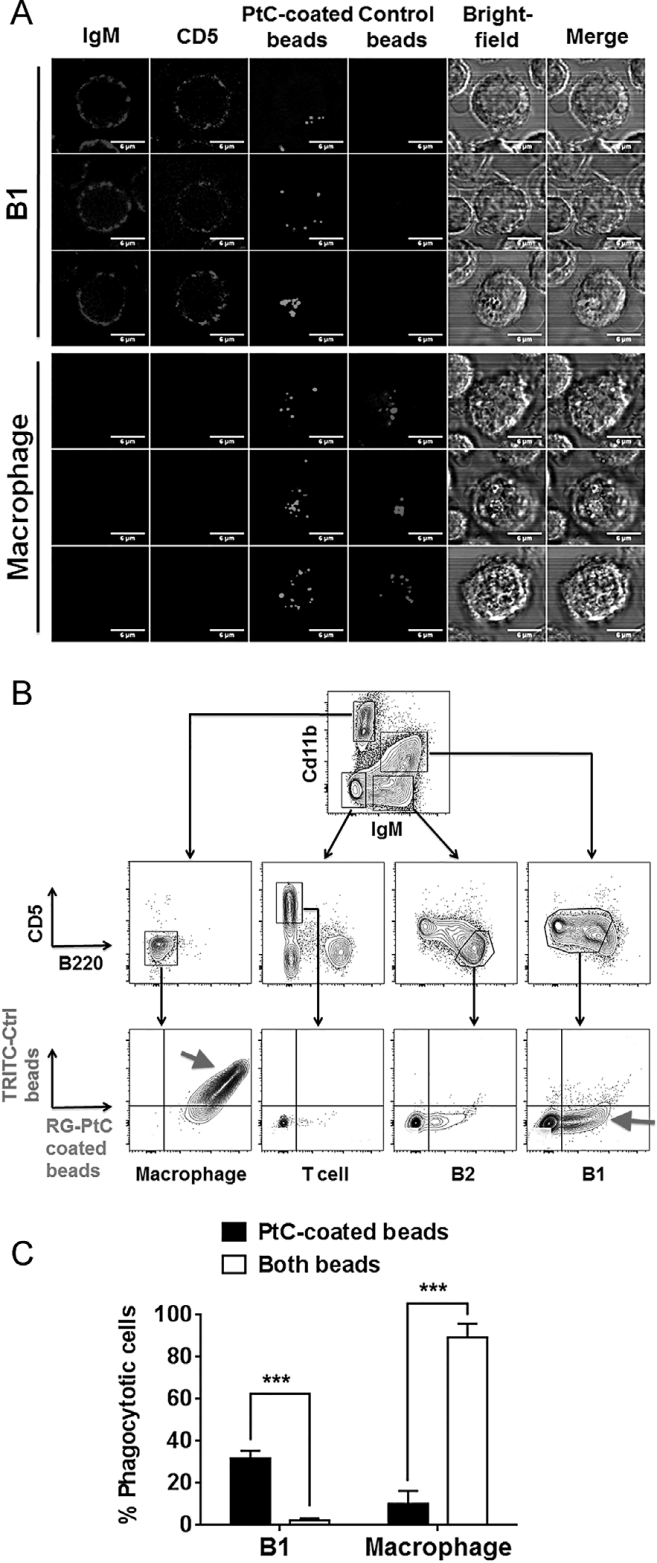
B1 B cells but not macrophages specifically phagocytose PtC beads. (A) PerC cells were incubated with Rhodamine green (RG)-PtC beads and TRITC (red)-control beads. Cells were then stained for IgM (blue) and CD5 (magenta). IgM^+^CD5^+^ B1 B cells were compared with IgM^−^CD5^−^ large macrophages for internalized red and green beads. (B) Representative flow cytometry gating of PerC cells after incubation with the beads. Red arrows indicate that most macrophages were double positive with red control beads and green PtC beads, whereas phagocytic B1 B cells were exclusively single positive with green PtC beads. (C) Percentages of B1 B cells and macrophages that have PtC-specific and non-specific (both beads) phagocytosis are compared. Results are mean ± SEM of 12 mice from 3 independent experiments.

After overnight co-culture of peritoneal cavity (PerC) cells with both PtC-coated and control beads, macrophages ingested more beads (10–30 cell^−1^) than B1 B cells (1–7 cell^−1^) (Fig. 1A). The larger phagocytosis capacity is likely contributed by the larger cytoplasm and cell size of macrophage. Interestingly, B1 cells exclusively ingested green PtC-coated beads, whereas macrophages indiscriminately engulfed both PtC beads and control beads (Fig. 1A). FACS analysis confirmed that phagocytic PerC B1 B cells exclusively ingested green PtC-coated beads (Fig. 1B–C), whereas, over 90% of macrophages phagocytosed both PtC and control beads (Fig. 1B–C). It is clear that compared to macrophages, B1 B cells displayed much higher specificity of phagocytosis toward PtC. B1 B cell phagocytosis may bridge the innate and adaptive immunity with this unique stringency and fulfill some physiological functions that conventional phagocytes could not achieve.

### High fat diet increases non-specific phagocytosis in B1 B cells

**Figure 2:**
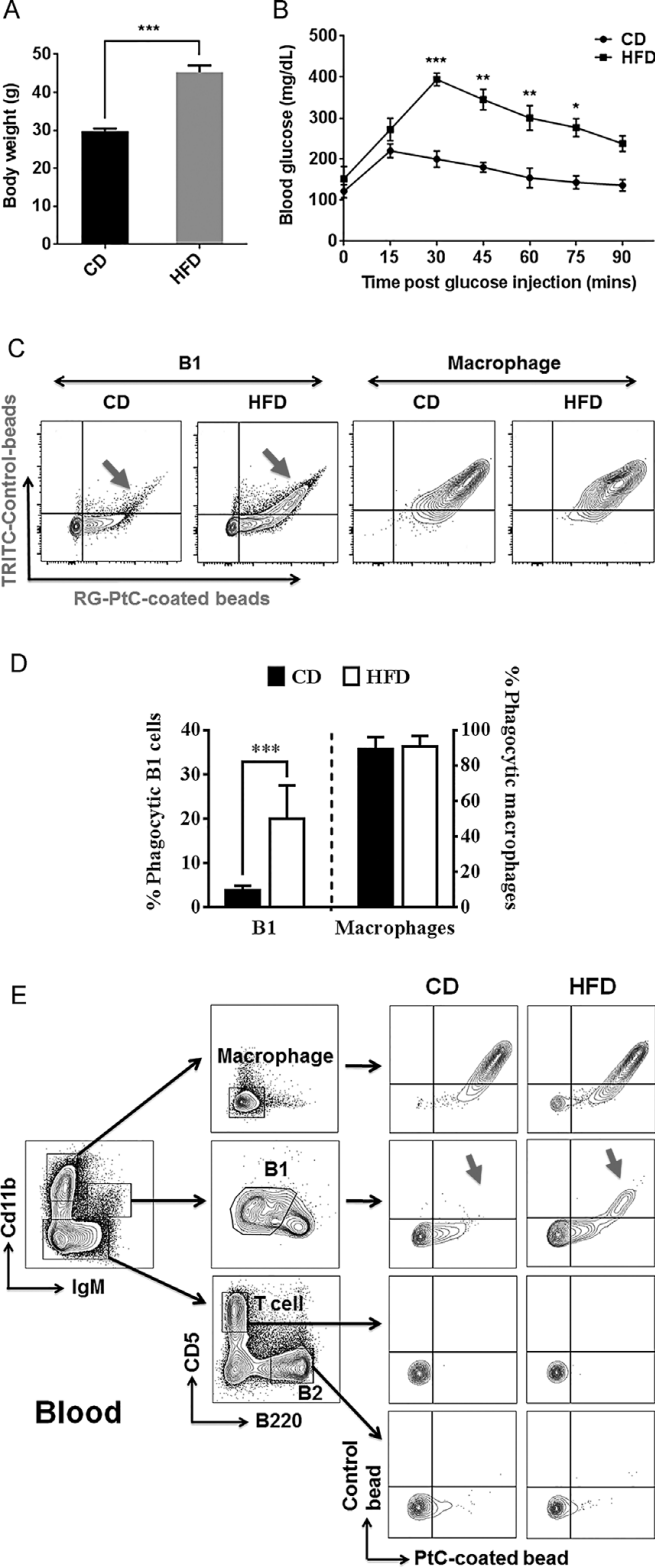
HFD increases non-PtC-specific phagocytosis by B1 B cells in DIO mice. Mice were kept on control diet (CD) and high-fat diet (HFD) for 16 weeks, body weight (A) and glucose tolerance (B) were measured in both groups. (C) FACS analysis of phagocytic cells from both CD and HFD groups. A significant increase of non-PtC-specific phagocytosis is observed in B1 B cells from HFD group as compared to CD group (red arrows). No significant change in macrophages was observed. (D) Percentage of phagocytic B1 B cells and macrophages with non-specific phagocytosis (engulf both PtC and control beads) in CD and HFD group (data are shown as mean ± SEM from *n* = 16 mice in each group). (E) Pooled peripheral blood lymphocytes from HFD and CD groups (*n* = 8) reveal the same increase of non-specific phagocytosis by B1 B cells (red arrows) in HFD group compared to those in CD group.

To test whether HFD affects the newly-discovered B1 B cell phagocytic function, peritoneal cells from HFD-fed, and control diet-fed mice were tested for phagocytosis. After 16 weeks of diet treatment, there was a 52% body weight increase (*P* < 0.0001) (Fig. 2A) and significant glucose intolerance developed in the HFD group (Fig. 2B). Peritoneal washout cells from both HFD and CD groups were harvested and incubated with a mixture of equal number of PtC-modified green fluorescent nano-particles and red fluorescent control particles. Peritoneal B1 B cells from CD group mice specifically phagocytosed green PtC-particles whereas B1 B cells from HFD group non-discriminately internalized both green and red particles (Fig. 2C, D).

While B1 B cells primarily reside in the pleural and peritoneal cavities in mice, they also circulate and are quickly mobilized during systemic events such as inflammation and infection 21. The human equivalent of murine B1 B cells were reported to be present in peripheral and umbilical cord blood by Griffin et al 22. Although controversy over the true identity of these human B1 B cells exists, many believe that these cells can be detected reliably in whole blood 23. Therefore, we examined whether HFD-induced changes in PerC B1 B cells could also be detected in blood. FACS analysis showed the same loss of specificity for PtC phagocytosis (red arrows) by blood B1 B cells in HFD-fed mice (Fig. 2E). Such correlation between PerC and blood further supports the notion that B1 cells circulate in response to systemic events such as obesity-induced inflammation.

**Figure 3:**
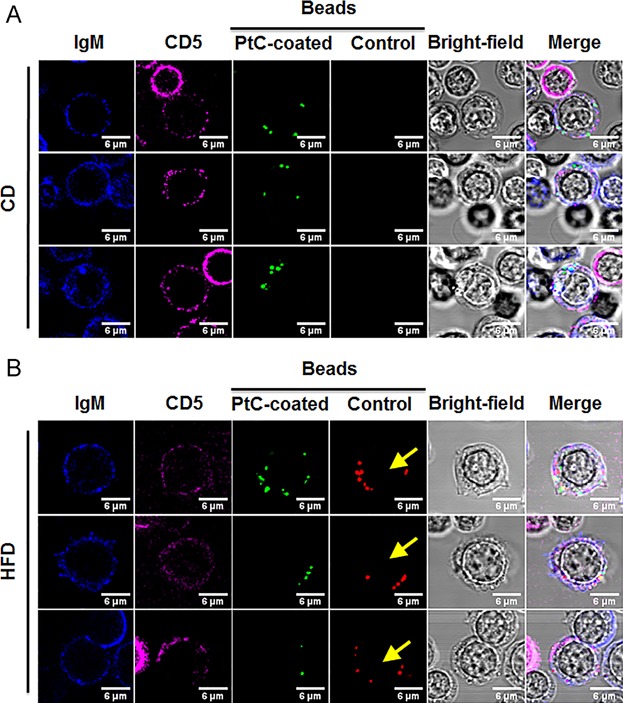
Microscopic analysis of B1 B cell phagocytosis from HFD and CD-fed mice. Peritoneal cells from CD group (A) and HFD group (B) were co-cultured with both PtC-coated and control beads and then stained for IgM (blue) and CD5 (magenta). B1 B cells are designated as IgM^+^CD5^+^ cells. Yellow arrows shown B1 B cells from HFD group took in control red beads, which is absent in CD group. Scale bars = 6 µm. Images are representative of three independent experiments.

This shift in specificity of phagocytosis for PtC was also captured by immunofluorescence microscopy (Fig. 3A, B), where B1 B cells from HFD groups took in both green and red beads (yellow arrows). B1 B cells in the HFD group not only had increased numbers of internalized beads, but also exhibited larger cytoplasm and cell size, resembling a B1-to-macrophage transition.

### Phagolysosome formation and actin polymerization are required by both B1 B cells and macrophage phagocytosis

**Figure 4:**
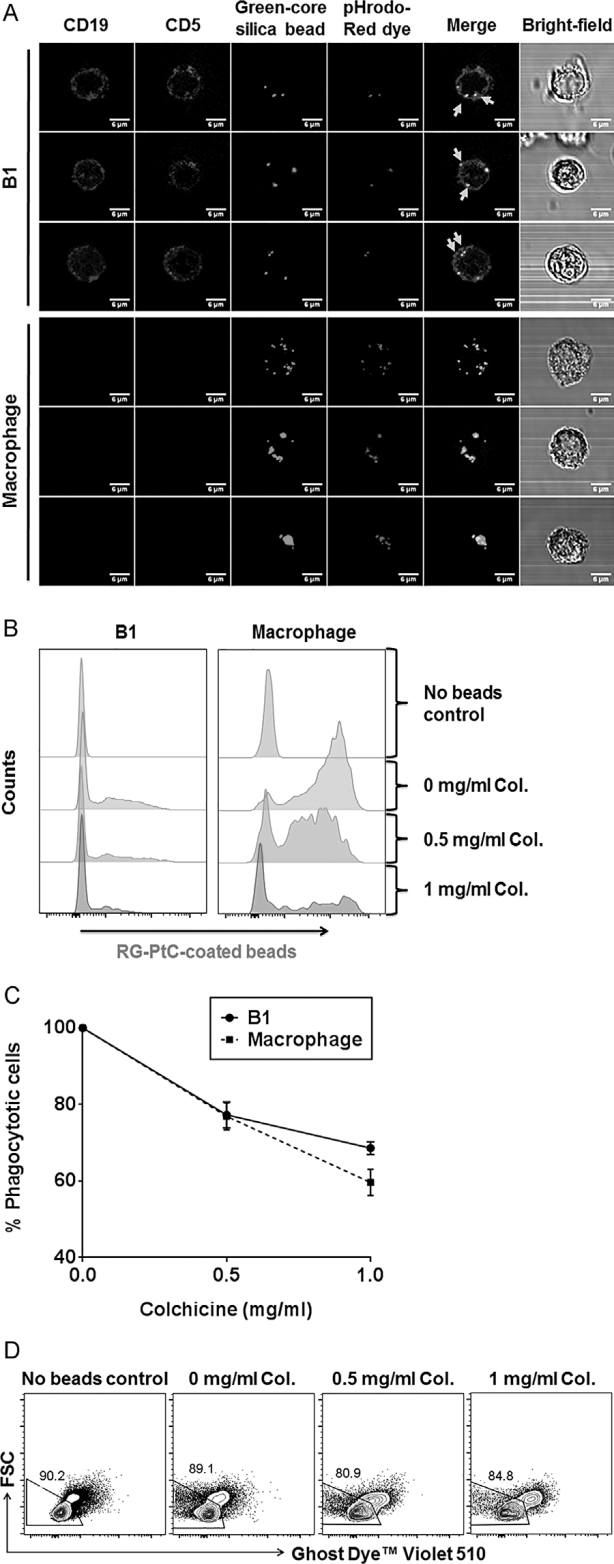
Phagolysosome formation and the requirement of cytoskeleton rearrangement during B1 B cell phatocytosis. (A) PerC cells were isolated from wild type C57BL/6 mice on normal diet. Representative microscopy images of phagocytic CD5^+^CD19^+^ B1 B cells and CD5^−^CD19^−^ macrophages ingesting green-fluorescent-core silica beads that were coated with PtC and pH-sensitive pHrodo-Red dye. Engulfed beads within phagolysosomes in B1 B cells emitted both red and green (merged as yellow) fluorescence (indicated by yellow arrows). Scale bars = 6 µm. (B) Peritoneal cells were incubated with PtC-coated beads and Colchicine solution with indicated concentrations overnight. Representative flow cytometry data showed decreasing green fluorescent with increasing doses of Colchicine. (C) Percentage of phagocytic B1 B cells and macrophages with Colchicine inhibition relative to that of untreated control sample (100%). Results are calculated as mean ± SEM from three independent experiments. (D) Live cell gating by Ghost Dye Violet 510 staining that excludes non-viable cells of the culture. Images were representative from three independent experiments.

In order to exclude the possibility that PtC beads were simply bound to surface IgM or absorbed into the cytosol, we used green fluorescent beads coated with PtC and pHrodo-Red to detect intracellular trafficking and phagolysosome formation. pHrodo-Red is a pH-sensitive fluorophore that increases fluorescence intensity when the environmental pH decreases from neutral to acidic. If the coated beads were absorbed by surface IgM and internalized into the cytosol, the pHrodo-red would not fluoresce in the neutral pH cytosol and only the pH-insensitive reference green fluorescence would be observed. If the beads were internalized through phagocytosis that involves lysosome fusion, both red pHrodo and green reference fluorescence would be detected and merged as yellow. Notably, our images revealed that beads internalized by a majority of the B1 B cells emitted both green and red fluorescence (Fig. 4A, yellow arrows), indicating that the beads were indeed located within acidic phagolysosomes.

Besides phagolysosome formation, the rearrangement of the cytoskeleton, which requires the involvement of actin filaments and microtubules, is also essential to phagocytosis 4,24. Therefore, disrupting the microtubules of the cytoskeleton can impair the phagocytic process. Colchicine is an anti-mitotic agent that binds to the end of microtubules and prevents their polymerization 25. Having an opposite effect to Colchicine, LPS stimulates polymerization of microfilaments and microtubules in macrophages and monocytes 26,27. Thus, we compared the effect of Colchicine on phagocytosis of PtC beads in the absence of LPS. B1 B cells can still phagocytose a significant amount of beads in the absence of LPS. FACS analysis showed that both macrophage and phagocytic B1 B cells treated with Colchicine experienced a reduction in phagocytic capacity in a dose-dependent manner (Fig. 4B, C). Such reduction was not due to toxicity of Colchicine as shown by FACS staining in which there was no significant increase of non-viable cells in the presence of various doses of Colchicine during the culture period (Fig. 4D).

## Concluding Remarks

Our study shows that B1 B cell phagocytosis differs from that of macrophages in that B1 B cells display selective phagocytosis of PtC-coated particles but not control particles. Supporting our finding, a recent report showed that PtC-modified liposomes are better adjuvants than other lipid-coated liposomes in promoting B1 cell phagocytosis and antigen presentation 28. Digestion of cell membranes by enzymes, such as phospholipase A2, renders the polar head group, PC moiety of PtC exposed to the external aqueous phase and thus accessible for recognition by B1 B cells 29. Such exposure is critical for non-inflammatory removal of senescent cells. It is reported that HFD alters phospholipid composition 30. It remains to be tested if such composition alteration causes the change in B1 cell phagocytosis. Recent studies also indicate that oxidized phospholipids within senescent or apoptotic cell membranes adopt conformations in which the oxidized lipids protrude into the aqueous phase 31,32. Interestingly, anti-PC IgM produced by B1 B cells also recognizes oxidized low-density lipoprotein (OxLDL) and provides protection from atherosclerosis 33. Thus, PtC-specific B1 B cell phagocytosis may play an important role in tissue homeostasis.

Previous studies indicate that HFD has adverse effects on the immune system 34,35. We show here that HFD induced a B1-to-macrophage transition, which might serve as a feedback mechanism to accelerate the clearance of various lipids or microvesicles released during adipose tissue inflammation. However, such response is at the cost of losing the critical phagocytic specificity for routine maintenance of tissue homeostasis.

Our study also suggests that phagocytic specificity of B1 cells can be a functional parameter in validating the human equivalent of murine B1 B cells. In addition, acquiring macrophage-like phagocytosis by B1 cells strongly suggests that B1 B cells could differentiate into phagocytes during inflammatory response 14,36. Further investigation is required to reveal the mechanism and physiological roles of such immune deviation and trans-differentiation in immune response to HFD-induced lipid overload and consequent inflammation.

## Material and Methods

### Mice

Male C57BL/6J mice were purchased from the Jackson Laboratory (Bar Harbor, ME). All procedures are approved by the Institutional Animal Care and Use Committee (IACUC) of Boston University Medical Center.

### Diet-induced-obesity (DIO) model and intra-peritoneal glucose tolerance test (IPGTT)

Six weeks old mice were fed with HFD (60% kcal fat, TD.06414, Harlan Laboratories) or control diet (18% kcal fat, 2918, Harlan Laboratories) for 16 weeks. Body weight was measured prior to IPGTT test. Mice were fasted for 6 h, followed by i.p. injection of d-glucose (G7528, Sigma–Aldrich) solution (1 g/kg body weight). Blood glucose levels were measured via tail blood using Contour glucose meter and test strips (Bayer, NJ) at 15-min increments.

### Preparation of phosphatidylcholine (PtC) and control beads

Fluorescent particles were prepared using a modified Stöber process 37. The bead size ranges from 500 to 600 nm. Dye precursor was prepared by reacting tetramethylrhodamine isothiocyanate (TRITC) dye with (3-aminopropyl)triethoxysilane (APTS). Rhodamine green beads were prepared in the same way except that a layer of PtC was spread on the bead surface 38.

### Preparation of green florescent beads with pHrodo-Red dye

Reverse phase evaporation was used to coat green fluorescent silica beads (Discovery Scientific, USA) with a lipid bilayer (inner leaflet: DOTAP; outer leaflet: 85 mol% PtC, 10% pHrodo-Red (P36600, Life Technologies) conjugated to DPPE, and 5% DSPE-PEG2k). The bead size distribution, concentration, and zeta potential were measured with qNano (Izon, Oxford, UK) and dynamic light scattering (90 Plus, Brookhaven). The final product had an average diameter of 530 nm.

### In vitro assessment of phagocytosis

Peritoneal cavity (PerC) cells were recovered by injecting HBSS with 2% FBS into the PerC. PBL were harvested from blood with cardiac puncture and RBC lysis. 1 × 10^6^ cells were incubated overnight at 37°C with fluorescent beads at 10:1 bead-to-cell ratio and 5 µg/mL LPS in supplemented RMPI medium in 96-well plates. Cells were harvested and subjected to confocal microscopy and flow cytometry analysis.

### Flow cytometry and confocal microscopy

Cells were incubated with Fc blockers (BioLegend) for 15 min, and then stained with the following antibodies for 45 min: B220-PerCp-Cy5.5, CD5-eF450, Cd11b-APC-eF780, IgM-APC, and Fixable Viability Dye eFluor® 506 (eBioscience) or Ghost Dye Violet 510 (Tonbo Biosciences) for live cell gating, CD5-AF647 (100614, BioLegend) and IgM-AF350 (Life Technologies) for microscopy. Cells were then washed three times by centrifugation. FACS data were collected with BD LSRII and analyzed using FlowJo software (TreeStar). Images were taken at 63X oil-immersion objective with Leica TCS SP5 microscope.

### Statistical analysis

Statistical differences were calculated by Student's *t*-test using GraphPad Prism software with *P* < 0.05 as statistical significance. (*P < 0.05, **P < 0.01, ***P < 0.001).
